# Women empowerment and skilled birth attendance in sub-Saharan Africa: A multi-country analysis

**DOI:** 10.1371/journal.pone.0254281

**Published:** 2021-07-07

**Authors:** Kwamena Sekyi Dickson, Kenneth Setorwu Adde, Edward Kwabena Ameyaw

**Affiliations:** 1 Department of Population and Health, College of Humanities and Legal Studies, University of Cape Coast, Cape Coast, Ghana; 2 Faculty of Health, School of Public Health, University of Technology Sydney, Sydney, NSW, Australia; University of Southern Queensland, AUSTRALIA

## Abstract

**Introduction:**

In 2017, the highest global maternal deaths occurred in sub-Saharan Africa (SSA). The WHO advocates that maternal deaths can be mitigated with the assistance of skilled birth attendants (SBAs) at childbirth. Women empowerment is also acknowledged as an enabling factor to women’s functionality and healthcare utilisation including use of SBAs’ services. Consequently, this study investigated the association between women empowerment and skilled birth attendance in SSA.

**Materials and methods:**

This study involved the analysis of secondary data from the Demographic and Health Surveys of 29 countries conducted between January 1, 2010, and December 3, 2018. For this study, only women who had given birth in the five years prior to the surveys were included, which is 166,022. At 95% confidence interval, Binary Logistic Regression analyses were conducted and findings were presented as adjusted odds ratios (aORs).

**Results:**

The overall prevalence of skilled birth attendance was 63.0%, with the lowest prevalence in Tanzania (13.8%) and highest in Rwanda (91.2%). Women who were empowered with high level of knowledge (aOR = 1.60, 95% CI = 1.51, 1.71), high decision-making power (aOR = 1.19, 95% CI = 1.15, 1.23), and low acceptance of wife beating had higher likelihood of skill birth attendance after adjusting for socio-demographic characteristics. Women from rural areas had lesser likelihood (OR = 0.53, 95% CI = 0.51–0.55) of skilled birth attendance compared to women from urban areas. Working women had a lesser likelihood of skilled birth attendance (OR = 0.91, 95% CI = 0.88–0.94) as compared to those not working. Women with secondary (OR = 2.13, 95% CI = 2.03–2.22), or higher education (OR = 4.40, 95% CI = 3.81–5.07), and women in the richest wealth status (OR = 3.50, 95% CI = 3.29–3.73) had higher likelihood of skilled birth attendance.

**Conclusion:**

These findings accentuate that going forward, successful skilled birth attendant interventions are the ones that can prioritise the empowerment of women.

## Introduction

More than three decades after the Safe Motherhood campaign was launched in Kenya [1], recent estimates show that 94% of all maternal deaths still occur in low and middle-income countries [2]. The highest global maternal deaths in 2017 was borne by sub-Saharan Africa (SSA), as nearly two-thirds of maternal deaths (196,000) occurred in the sub-region. This notwithstanding, a 40% decline in maternal deaths in SSA occurred between 2000 and 2017. To expedite the reduction in maternal deaths, WHO accentuates that utilising the service of a skilled birth attendant (SBA) at delivery is indispensable [2]. Plethora of empirical evidence supports the position of WHO on the essence of skilled care at birth in saving the lives of women and newborns [3,4].

A multitude of factors interplay in women’s ability to obtain the assistance of SBAs in SSA. Previous country-specific studies across SSA have reported issues such as low education and low wealth status as key barriers [5,6]. These structural and contextual factors have resulted in in-country and inter-country variations in utilisation of the service of SBAs [7,8]. To have a better understanding of barriers and facilitators of maternal healthcare utilisation in general, there have been some country-level studies across SSA [9,10]. For instance, evidence from Ghana and Kenya indicate that antenatal visits are generally high relative to the proportion of women who seek the services of SBA [11,12]. A number of factors contribute to the situation.

Women empowerment is one of such factors and has been acknowledged as an enabling factor to women’s functionality and healthcare utilisation. With time, women empowerment has been conceptualised with varied indicators. It is sometimes referred to as women’s autonomy [13,14], whilst some authors have attempted to distinguish between the two concepts [15,16]. Largely, several authors converge that women empowerment is measured with indicators such as decision making competencies [14,17,18], educational attainment or depth of knowledge [19–21], the reaction towards a partner or domestic violence such as wife beating [22,23] and labour force participation [18,24].

Consistent evidence indicates that empowered women have high maternal healthcare utilisation including the services of SBAs [25–28]. Most of these studies were conducted outside SSA, whilst those within the sub-region were conducted within some specific countries such as Senegal and Tanzania [29], as well as Eritrea and Ethiopia [30]. Conclusions and recommendations from these studies cannot be generalised to the SSA region in entirety and hence the need for a study to ascertain the magnitude and direction of how women empowerment relates with skilled birth attendance in SSA, hence the need for this study. Using four empowerment indicators (decision making, knowledge level, labour force participation, and acceptance of wife beating), this study, therefore, seeks to assess the relationship between women empowerment and skilled birth attendance in SSA.

## Materials and methods

### Ethical approval

Questionnaires and procedures for the surveys were reviewed and approved by the Ethics Committee of Opinion Research Corporation Macro International Inc and ICF Institutional Review Board (IRB). As nationally representative surveys, the DHS survey protocols for the various countries were also reviewed and approved by the ICF IRB and the relevant IRBs of the various countries. All data were completely anonymized, de identified, and/or aggregated before access and analysis. Detailed information on the ethical procedures observed by the DHS program can be accessed via http://goo.gl/ny8T6X. As we used secondary data for our analysis, we did not require further ethical approval from our named institutional bodies as the national level ethical clearance was sufficient for our analysis to be carried out.

### Data

The study used pooled data from the most recent Demographic and Health Surveys (DHS) conducted between January 1, 2010, and December 3, 2018, in 29 countries in sub-Saharan Africa (SSA) (see Table 1). The DHS is a countrywide representative study undertaken in a five-year period in several low–and middle–income countries in Asia and Africa. It focuses on maternal and child health by interviewing women in their reproductive age (15–49 years). The DHS follows standardized procedures in areas such as sampling, questionnaires, data collection, cleaning, coding, and analyses, which allow for comparability across countries. For this study, only women who had given birth in the five years prior to the surveys were included, which is 166,022.

**Table 1:** Participant number, % and SBA practices in different countries in SSA.

Country	Frequency	SBA (%)	95%CI for SBA
Benin	7,678	78.1	3.51(3.32, 3.70)
Burundi	7,812	85.9	6.07(5.69, 6.47)
Cameroon	5,050	67.6	2.32(2.18, 2.47)
Chad	2,891	67.2	0.28(0.26, 0.31)
Comoros	1,416	85.3	5.39(4.66, 6.23)
Congo DR	8,645	81.0	2.93(2.79, 3.07)
Cote d’voire	3,954	60.8	1.34(1.26, 1.42)
Ethiopia	6,926	30.3	0.57(0.54, 0.60)
Gabon	2,057	90.3	3.72(3.37, 4.10)
Ghana	3,357	75.3	2.58(2.40, 2.78)
Gambia	4,591	54.9	1.50(1.41, 1.58)
Guinea	4,790	54.9	1.16(1.09, 1.22)
Kenya	5,329	66.6	1.46(1.39, 1.5)
Lesotho	1,933	80.8	3.63(3.26, 4.04)
Liberia	3,239	62.9	1.35(1.27, 1.44)
Malawi	10,926	90.4	10.89(10.18, 11.66)
Mali	5,956	69.2	1.96(1.86, 2.07)
Mozambique	5,780	55.7	1.62(1.54, 1.71)
Namibia	1,273	86.2	0.78(0.76, 0.81)
Niger	7,354	32.0	0.63(0.60, 0.66)
Nigeria	19,922	44.4	0.78(0.76, 0.81)
Rwanda	4,716	91.2	10.76(9.70, 11.93)
Sierra Leone	5,960	91.0	1.58(1.50, 1.66)
Senegal	5,860	59.3	1.42(1.35, 1.49)
Tanzania	5,499	13.8	0.14(0.13, 0.15)
Togo	4,159	45.5	0.62(0.58, 0.65)
Uganda	7,911	75.6	2.91(2.77, 3.06)
Zambia	6,886	67.7	2.13(2.02, 2.24)
Zimbabwe	4,073	67.7	5.09(4.68, 5.54)
All Countries (total)	166,022	63.0	

### Description of variables

#### Outcome variable

The main outcome variable was skilled birth attendance. The outcome variable was derived from the response to the question "who assisted with the delivery?" Responses were categorized under health personnel ‘1’ and other persons ‘0’. Health personnel included doctor, nurse, nurse/midwife, an auxiliary midwife; other person also consisted of a traditional birth attendant (TBA), traditional health volunteer, community/village health volunteer, neighbours/friends/relatives, other. For this study, skilled birth attendance referred to births assisted by a doctor, nurse, auxiliary midwife, nurse/midwife [4].

#### Explanatory variable and covariates

Women empowerment was the main explanatory variable. The elements of women empowerment consisted of; 1. labour force participation (working, not working); 2. acceptance of wife beating (neglect of a child, burning of food, arguing with husband/partner, refusal to have sex with husband/partner, going out without permission); 3. decision making power (this was measured by the person who decides for respondents’ health care, house earning and household purchase and visiting family members); and 4. knowledge level (comprising listening to radio, reading newspaper/magazine, watching television, and educational level). Decision making power, knowledge level and acceptance of wife beating were coded based on previous methodology [31]. This is in accordance with the methods of previous authors [31,32].

Nine other explanatory variables or covariates were included namely: age, residence, partner’s level of education, wealth status, number of antenatal care (ANC) visits, skilled ANC provider, getting medical help for self: money needed for treatment, distance to a health facility and getting permission to go. These explanatory variables were selected due to their positive association with skilled birth attendance as found by prior studies [4,8,33]. Age was grouped in 5 –year interval and captured as 15–19 = 1, 20–24 = 2, 25–29 = 3, 30–34 = 4, 35–39 = 5, 40–44 = 6, and 45–49 = 7. Residence was categorized as urban = 1 and rural = 2. Women and partner’s levels of education were captured as no education = 1, primary = 2, secondary = 3, and higher education = 4. Wealth status was categorized as poorest = 1, poorer = 2, middle = 3, richer = 4, and richest = 5. Marital status was captured as married = 1, cohabitation = 2, widowed = 3, divorced = 4 and separated = 5. The number of Antenatal Care (ANC) visits was captured as less than four visits = 1 and four or more visits = 2. Skilled ANC provider was categorised as no = 0 and yes = 1. Getting medical help for self: money needed for treatment, distance to a health facility, and getting permission to go were captured as a big problem = 1 and not a big problem = 2.

### Data analysis

Descriptive and inferential analyses were done. The descriptive analysis reported results on the four elements of women empowerment, explanatory variables, and the country specific, and pooled prevalence of skilled birth attendance in sub-Saharan Africa. Inferential analysis was used to explore the relationship between skilled birth attendance, women empowerment, and the covariates. Binary Logistic Regression was conducted. All results of the binary logistic analyses were presented as odds ratios (ORs) and adjusted odds ratios (aORs) with 95% confidence intervals (CIs). All analyses were done using Stata version 14. The complex nature of the sampling structure of the data was adjusted using the Stata Survey command ‘svyset v021 [pweight = wt], strata (v023)’.

## Results

### Participant number, % and SBA practices in different countries in SBA

With skilled birth attendance average of 61.2%, the highest occurred in Rwanda (91.2%) whilst the least was observed in Tanzania (13.8%) as shown in Table 1.

### Background characteristics, and skilled birth attendance

Women aged 20–24 years commonly had skilled birth attendance (63.8%). Eight out of ten women from urban areas used the service of SBAs (82.9%). Among the richest group 89.1% of the women had skilled birth attendance. Skilled birth attendance was more prevalent among women whose partners had higher education (86.7%), had four or more ANC visits (73.5%) or had skilled ANC provider (72.5%) (see Table 2). The study revealed that 68.8% of the women who reported that getting money for treatment for medical help for themselves was not a problem had skilled birth attendance. Similarly, 69.9% of those who indicated that distance to health facility was not a big problem, and 62.7% of those who had no problem with seeking healthcare had skilled birth attendance. Also, 77.2% women who listened to radio almost every day, and 91.7% who watched television almost every day had skilled birth attendance (see Table 2).

**Table 2:** SBA practices according to the background characteristics of participants.

Variable	Frequency (N = 166,022)	Proportion of SBA (row percentage)	P value	Unadjusted OR [95%CI] for SBA
***Age***			0.000	
15–19	9,766	60.1		Ref
20–24	34,533	63.8		1.14***(1.09, 1.20)
25–29	43,383	63.4		1.12***(1.07, 1.17)
30–34	35,387	63.5		1.13***(1.08, 1.18)
35–39	25,741	60.8		1.01(0.96, 1.06)
40–44	12,770	57.0		0.86***(0.81, 0.91)
45–49	4,442	51.7		0.70***(0.65, 0.75)
***Place of residence***			0.000	
Rural	117,244	53.5		Ref
Urban	48,778	82.9		0.24***(0.23, 0.25)
***Wealth status***			0.000	
Poorest	39,977	41.9		Ref
Poorer	35,466	53.3		1.58***(1.54, 1.63)
Middle	33,026	61.9		2.25***(2.18, 2.32)
Richer	30,115	75.1		4.18***(4.04, 4.32)
Richest	27,438	89.1		11.35***(10.87, 11.85)
***Partner’s educational level***			0.000	
No education	58,144	44.7		Ref
Primary	49,894	63.9		2.19***(2.14, 2.25)
Secondary	46,184	75.8		3.87***(3.76, 3.97)
Higher	11,800	86.7		8.04***(7.60, 8.50)
***Number of ANC visits***			0.000	
Less than four	74,963	48.3		Ref
Four or more	91,059	73.5		2.97***(3.03)
***Skilled ANC provider***			0.000	
No	28,998	13.3		Ref
Yes	137,024	72.5		17.15***(16.54, 17.78)
***Getting medical help for self*: *money needed for treatment***			0.000	
Big problem	88,101	56.3		Ref
Not a big problem	77,921	68.8		1.59***(1.55, 1.63)
***Getting medical help for self*: *distance to health facility***			0.000	
Big problem	68,607	51.0		Ref
Not a big problem	97,415	69.9		2.23***(2.19, 2.28)
***Getting medical help for self*: *getting permission to go***			0.000	
Big problem	28,407	52.8		Ref
Not a big problem	137,615	64.0		1.17***(1.68, 1.75)

P<0.10 * p<0.05 ** p<0.01*** Ref = Reference category.

We found that women from rural areas had a lesser likelihood (OR = 0.51, CI = 0.49, 0.53) of skilled birth attendance compared to women from urban areas (see Table 2). It was also observed that there were higher odds of skilled birth attendance for women in richest wealth status (OR = 3.67, CI = 3.44, 3.92) compared to those with poorest wealth status. Women whose partners had higher education were more likely (OR = 1.56, CI = 1.44, 1.69) to utilise the service of the service of SBA compared with those whose partners had no education (see Table 2).

Women who had four or more antenatal care (ANC) visits had a higher likelihood (OR = 1.69, CI = 1.64, 1.74) of skilled birth attendance compared to those who had less than four antenatal care visits (see Table 2).

#### Women empowerment and skilled birth attendance in sub-Saharan Africa

At least six in ten women who reported having high decision-making power (70.0%), high level of education (81.6%), working (64.0%) and low acceptance of wife beating (67.8%) had skilled birth attendance during their delivery (see Table 3).

**Table 3:** Predictors of skilled birth attendance utilization among women in sub-Saharan Africa.

Variable	Frequency (N = 166,022)	Proportion of SBA	P value	Unadjusted OR	Adjusted OR^1^
**Decision-making power**					
Low	43,938	51.3	0.000	Ref	Ref
Medium	54,091	61.1		1.15***(1.12, 1.18)	1.07***(1.03, 1.10)
High	67,993	70.0		1.53***(1.49, 1.57)	1.19***(1.15, 1.23)
**Women’s knowledge level**					
Low	33,220	35.9	0.000	Ref	Ref
Medium	91,441	62.8		2.67***(2.62, 2.76)	1.25***(1.20, 1.30)
High	41,361	81.6		6.20***(5.98, 6.41)	1.61***(1.51, 1.71)
**Labour force participation**					
Not working	46,717	61.2	0.000	Ref	Ref
Working	112,110	64.0		1.01(0.99, 1.03)	0.91***(0.88, 0.94)
**Acceptance of wife beating**					
Low	100,083	67.8	0.000	Ref	Ref
Medium	31,106	59.1		0.76***(0.74, 0.78)	0.89***(0.86, 0.92)
High	34,833	48.4		0.57***(0.56, 0.59)	0.81***(0.77, 0.83)

^1^Adjusted for background characteristics and country p<0.10 * p<0.05 ** p<0.01***.

We found that women with high decision-making power had higher likelihood (aOR = 1.19, CI = 1.15, 1.23) of skilled birth attendance compared to women with low decision-making power after adjusting for socio-demographic characteristics. Also, women with high level of knowledge had higher odds (aOR = 1.61, CI = 1.51, 1.71) of utilizing the service of SBA compared with women with low level of knowledge after adjusting for socio-demographic characteristics (see Table 3). Women with high acceptance of wife beating had a lesser likelihood (aOR = 0.81, CI = 0.77, 0.83) of skilled birth attendance during delivery compared to those with low acceptance of wife beating after adjusting for socio-demographic characteristics (see Table 3).

## Discussion

The study examined the prevalence of skilled birth attendance and the association between women empowerment and SBA in 29 SSA countries. We found that the overall prevalence of skilled birth attendance in SSA was 63%. However, this varied from 13.8% in Tanzania to 91.2% in Rwanda. This shows that although utilisation of the services of SBAs in SSA appears high, it is very low in some individual countries. We also found that women aged 20–24 years and women in urban areas highly utilise the services of SBAs. The study found that utilisation of SBA services was highest among women who were empowered (i.e. those with high decision-making power, high level of knowledge, working and have low acceptance of wife beating). This affirms the findings of Khatiwada et al. [27] and that of Ameyaw et al. [33] that women with decision-making autonomy, media and information empowerment have a high utilisation of SBA.

Realising that women with high level of knowledge were more likely to utilise the services of SBA as compared to their counterparts with low level of knowledge affirms previous studies, which found that maternal knowledge and education have significant and positive relationship with skilled birth attendance [3,19,20,34,35]. With higher knowledge, the chances of a woman to be more knowledgeable about the need and importance of having a skilled birth attendance advances [5,6]. Education empowers women to have autonomy over their bodies and make informed decisions. Ameyaw et al. [33] argued that women with autonomous health decision-making power were more likely to utilise health facilities for delivery as compared to their counterparts without it. This affirms the findings of the current study that women with high decision-making power are more likely to utilise the services of SBA as compared to their counterparts with low decision-making power.

Women with high acceptance of wife beating had a lesser likelihood of SBA utilisation during delivery which affirms the argument of Khan and Islam [36] that women’s attitude towards wife beating have a significant effect on the reproductive health care seeking behaviour of women. A probable explanation is that this category of women lack entitlement and empowerment [37] to negotiate a safer reproductive health decision or making healthcare seeking decision.

It was further observed that having one’s partner attained a higher level of education increases the likelihood of a woman utilising SBA. Women whose partners had higher education, being more likely to utilise the service of skilled birth attendants is consistent with Bhowmik et al. [38]. Our finding is probable because most SSA countries are largely patriarchal societies [39,40], hence partners with high education who are aware of the importance of skilled birth attendance can easily induce their wives to utilise the service of SBAs.

Women in rural areas were less likely to utilise the service of SBAs compared to their counterparts in urban areas. Alemayehu and Mekonnen [35] as well as Ayele et al. [34] reported similar results that women who reside in urban areas are more likely to utilise the services of SBAs compared to their rural counterparts. Geographically, urban areas tend to have easy access to skilled birth attendance compared to rural areas and this may enhance the likelihood of urban dwellers having skilled birth attendance than those in rural areas [41]. In Ghana, for instance, urban and relatively developed locations have increased availability and access to health facilities and services as compared to rural areas [42] and this may account for the observed variation.

Women with richest wealth status were more likely to utilise the service of SBAs. This finding corroborates that of Atuoye et al. [43] where wealth disparity was observed as a determiner in the utilisation of SBAs’ services. The possible explanation may be that women within the richest wealth status will be able to pay for the services of SBAs compared with the poor [4,34].

Women who patronised skilled ANC providers were more likely to utilise the service of SBAs compared to those who did not use the services of a skilled ANC providers. Similarly, women who had four or more ANC visits were more likely to utilise the service of SBAs as compared to their counterparts with less than four ANC visits. This finding is similar to other studies which also found that frequency of visit to ANC significantly associates with utilisation of SBAs’ services [34,44,45]. A possible explanation to this is that women who patronise ANC are likely to receive some education on the importance of utilising the services of SBAs during their visits to ANC [3].

It was also observed from the study that women who do not have a big problem with distance to health facility were more likely to utilise the services of SBAs as compared to their counterparts who have a big problem with distance to health facility. This finding coincides with the findings of Ameyaw and Dickson [4] who inquired on SBA utilisation in Sierra Leone, Niger and Mali. This alludes to the fact that distance to health facilities has a major role in the health seeking behaviour of women [46].

## Strengths and limitations

This study employs a rigorous analytical approach in investigating the underlying factors of SBA in SSA. We used large, representative datasets of 29 SSA countries and these strengthen the validity and generalisability of our findings. These notwithstanding, the study has some shortcomings. First, the cross-sectional design of the study does not allow causal inference between the socio-demographic factors and SBA. Second, depending on the social and neighbourhood factors of the women, there is a possibility of social desirability bias in the women’s responses. Thus, a woman living in a community that lauds SBA may indicate that she had skilled birth attendance, which may not necessarily be the reality.

## Conclusion

Women empowerment (i.e. decision-making power, level of knowledge, labour force, acceptance of wife beating), place of residence, age, partners educational level, skilled ANC providers, number of ANC visits, and getting medical help for self: distance to health facilities, were noted to have a significant association with SBA utilisation. These findings accentuate that future successful SBA interventions are the ones that prioritise the empowerment of women and interventions to empower women to negotiate safer maternal and reproductive health care decisions. The SBA prevalence in Tanzania can be enhanced by understudying countries with high SBA, such as Rwanda, in order to adopt their prevailing successful interventions whilst taking cognisance of contextual variations.
